# Strength training intervention for hybrid workers: a randomised pilot feasibility trial

**DOI:** 10.1038/s41598-025-27567-9

**Published:** 2025-11-25

**Authors:** Christopher D. Connelly, Stuart Gray, Nur Dania Rosaini, Gemma C. Ryde

**Affiliations:** 1 School of Sport, Exercise and Health Sciences, Loughborough University, Loughborough, England; 2 School of Cardiovascular and Metabolic Health, University of Glasgow, Glasgow, Scotland

**Keywords:** Workplace, Health promotion, Resistance training, Stress, Productivity, Work engagement, Physiology, Public health, Quality of life

## Abstract

There is a major productivity and economic loss due to poor mental and physical health of the workforce. These factors can be improved by muscle strengthening exercise, which few people currently do. This study investigated the effect of a simple, time-efficient, resistance band training intervention on physical function and work-related outcomes in hybrid-working employees. In this pilot randomised controlled trial, untrained hybrid-working employees were randomised to a four-week resistance band training intervention (15 min 3x per week) or control group. Written and video instructions on the exercises were provided. The primary outcomes were physical function; 30s sit to stand and 30s push-up test, with secondary outcomes of perceived stress, work engagement and productivity. Outcome data was collected via an online survey. Fifty participants (age 46 ± 11 years; 41 female) were recruited to the study, with 46 completing the trial. Compared to the control group, the intervention group (adherence 89%) saw significant improvement in sit-to-stand (mean difference 5.34 ± 7.88 (SD), 95% CI [1.94, 8.75]; p = 0.04) and 30s push-up (mean difference 5.00 ± 3.37 (SD), 95% CI [3.54, 6.46]; p < 0.001) test score. Significant improvements were also found for perceived stress (p = 0.002), total work engagement (p = 0.008) and total productivity (p = 0.004). Four weeks of a simple, time-efficient resistance band training intervention improved lower and upper body physical function, stress, work engagement and productivity. This intervention may be a practical and simple strategy to improve adherence to muscle strengthening guidelines in hybrid working employees, whilst improving physical health and work-related outcomes.

**Supplementary Information:**

The online version contains supplementary material available at 10.1038/s41598-025-27567-9.

## Introduction

Since the 2019 pandemic, working practices for many former office-based employees have changed significantly. In most countries, working from home increased during this time, with data from the UK suggesting 5.7% of employees were working from home at the beginning of 2020, increasing to between 43.1% and 46.6% in April 2020 during the pandemic^1,2^. Data from the UK Office for National Statistics suggests that working from home in some form is set to stay, with 85% of employees reporting they would like to continue a hybrid approach to working (part at home, part in a place of work) with other countries such as the US suggesting this is the case for 47% of employees^3,4^. Similarly, many employers have reported no plans to return all staff to offices full time in the future, with reports suggesting up to 70% of employers will continue with a hybrid working approach^3^. Hybrid working, therefore, seems set to continue for many working-age adults globally.

This change in working practices has however, affected employees’ lifestyle behaviours and health and wellbeing, although the relationships are complex. A UK omnibus survey of over 2000 employees showed positive benefits from working from home, with 47% of respondents suggesting it has improved their wellbeing, and 78% suggesting improved work-life balance^5^. However, in a rapid review of 23 papers on the mental and physical effects of working from home, negative outcomes such as increased stress and difficulties switching off from work were reported^6^. Similar complex relationships have also been reported for lifestyle behaviours such as physical activity, with some individuals reporting an increase in total physical activity, some a decrease, and others staying the same but switching the domains in which physical activity occurs (for example, doing more leisure time activity but less active transport)^7^.

Less is known about how a key ‘forgotten’ guideline of physical activity, muscle strengthening exercise^8^, has changed as a result of hybrid working. Prior to the pandemic in 2018, whilst most UK adults reported meeting the physical activity guideline for moderate to vigorous physical activity (66% in Scotland, for example), fewer (29%) met the guidelines when two days of muscle strengthening exercise per week were included^9^. There is limited data on how the increase in working from home has influenced employees’ ability to meet the muscle strengthening exercise guidelines. Regardless, there is an opportunity to promote strength training in this new workplace setting to those who might not have engaged in more conventional strength settings, such as gyms or felt comfortable exercising in an office setting.

To date, very few home-based strength interventions for employees have been trialled, but effects on physical health and strength outcomes from other populations are largely positive. Most literature on home-based strength interventions has focused on older adults or clinical groups and has been shown to be effective. For example, a meta-analysis of home-based strength interventions including 17 randomised controlled trials and 1,477 healthy older adults showed small positive effects on muscle strength, power and endurance^10^. There is further evidence on the types of strength training that might be suited to hybrid working employees, such as resistance bands, but these haven’t been explicitly tested in hybrid workers. A meta-analysis of eight trials using resistance bands compared to conventional strength equipment (machine weights and dumbbells) in a very diverse range of populations (including patients with coronary heart disease, football players, and physically fit females) reported that resistance band training resulted in similar upper and lower limb strength gains to conventional strength equipment^11^. Combined, these results suggest home-based interventions and resistance bands hold considerable promise.

It remains essential to establish the effectiveness of these interventions within the specific context of the workplace and home-working environments. Qualitative evidence indicates that workplaces present unique barriers to physical activity, including high workload, cultural and normative factors (“no break” cultures), and organisational concerns (productivity loss)^12^. Whilst home-based resistance band interventions may circumvent some of these barriers (home settings provide a private, ‘non-work’ space for activity), other barriers, such as time constraints and competing demands, may persist. Accordingly, interventions that target outcomes meaningful to employees and organisations, such as stress (a leading cause of workplace absence), productivity, and work engagement, are especially important for the uptake and sustainability of workplace health interventions. In addition to these pragmatic reasons, empirical evidence suggests that physical activity, including strength training, can influence stress^13^ and work-related outcomes through interconnected physiological and psychosocial pathways. For stress, strength training can have positive adaptations to the HPA Axis and cortisol levels^14^, promoting the release of endorphins and other neurochemicals^15^, which are associated with the stress response. Beyond physiological effects, strength training is associated with improvements in self-efficacy, body image, and perceived control^16^, - psychological resources that buffer against stress and facilitate adaptive coping. Whilst there is less evidence on strength training and work-related outcomes, physical activity can positively influence productivity through increased vigour^17^ and capacity in performing work tasks^18^, which may contribute to greater productivity at work.

There is a need for home-based muscle strengthening interventions tailored to hybrid working employees, with evaluation of outcomes that support their long-term adoption and sustainability within workplaces. The aim of this study was to investigate the effect of a time-efficient, resistance band training intervention for hybrid working employees on physical function and work-related outcomes.

## Methods

### Trial design

The study was a 4-week, parallel group, two-arm randomised controlled trial with waitlist control, undertaken between June 2022 to July 2022 in the United Kingdom. This trial is reported in line with the CONSORT statement of reporting randomised controlled trials^19^ and registered at the Protocol Registration and Results System (Clinicaltrias.gov Ref: NCT07086885, 18/7/2025). Hybrid-working adults were randomised to a resistance band training intervention group or wait-list control group, and the impact of the intervention on physical function tests and work-related outcomes was assessed. Ethical approval was granted by The University of Glasgow College of Medicine, Veterinary & Life Sciences Ethics Committee (Ref: 200210134, 20/05/2022) and was performed in accordance with the Declaration of Helsinki.

### Participants and randomisation

Participants were recruited via emails to previous workplace contacts of the research team from around the UK and included mainly local government authorities, NHS workplace health units and universities. The study was also advertised via social media channels (Facebook, Twitter and blog posts) and speculative emails, particularly to Scottish-based businesses.

Eligible participants were adults aged 16+, working from home in the United Kingdom on 3 or more days per week, performing less than 2 days per week of muscle strengthening exercise as defined by the UK physical activity guidelines, were not on holiday during the study or answered yes to any of the questions in the Physical Activity Readiness Questionnaire for Everyone (PAR-Q+)^20^. After initial screening for these eligibility criteria via response to an email, participants were provided with an information sheet and all participants provided informed written consent. Randomisation was performed in Microsoft Excel using random number generation into two equal groups (simple randomisation) after completion of baseline testing. Author CC conducted the randomisation, enrolment of participant’s and assignment to intervention or control group. The participants were not involved in the design, conduct, reporting, or dissemination plans of this study.

### Resistance band intervention group

Participants received a set of fabric looped resistance bands (Elvire worth £14.99). The set consisted of 3 bands of different levels of resistance, from easy, medium, to hard. Participants were provided with both written and video instructions on how to use the bands and perform the exercises.

The home-based resistance band training intervention was performed 3 times per week for 4 weeks, and the session duration was around 15 min. Each session consisted of four exercises (Table 1), which were completed one after the other. Upper and lower body exercises were alternated so that as one muscle group was resting, another was being used, which has been recommended particularly in untrained individuals who may find continuous upper or lower body exercises too difficult^21^. Participants were guided to complete an 8–12 rep range per exercise. If they completed 12 reps in all sets, then in the next session, they were advised to upgrade to a harder exercise variation or a band of greater resistance. If they could not complete 8 reps, then they were asked to reduce the intensity (easier variation or lower band). In weeks 1 and 2, participants completed 2 sets of each exercise, and this increased to 3 sets in weeks 3 and 4. Participants were recommended to keep rest minimal between sets (0–20 s) but could take up to a minute if necessary.

**Table 1:** Intervention exercises with Variations.

Exercise	Variations
Squat	1.Sit to Stand 2. Squat 3. Reverse Lunge
Push Up	1. Standing, pushing from an elevated surface (e.g. Kitchen counter) 2. Push-up with knees on the ground 3. Standard push-up
Deadlift	n/a
Seated row	n/a

Deadlift and Seated Row performed using resistance bands, squat and push up variations performed using bodyweight or banded. n/a = not applicable.

### Control group

Those in the control group were asked to maintain their usual physical activity throughout the intervention. They were provided with the intervention resources to complete on their own after the 4 weeks.

### Outcomes

All data was reported using Jisc Online Surveys (https://www.onlinesurveys.ac.uk/ University of Glasgow licence), including demographic information, primary (Strength: physical function tests) and secondary outcomes (Work-related: stress, work engagement, and productivity) are detailed further below. The baseline survey was completed in the week before the 4-week intervention period, and the final survey was completed the week after the intervention. All physical function tests were collected in the employees’ own homes with instructional videos on how to perform the physical function tests provided before completing the tests. The researcher was not present during testing.

#### Demographic data

Data were collected on year of birth, gender, ethnicity, postcode, education, employment status, income, work sector, hours worked and perceived health.

#### Physical function tests

Participants were instructed to set a stable chair against a wall and set a timer for 30 s, then perform as many full repetitions as possible from seated to standing upright. The participants performed this with their arms on the opposite shoulder so they could not use them to swing or push themselves up from the chair.

Participants were instructed to perform as many push-ups as possible in 30 s, ensuring they maintained a rigid, straight body shape, fully extending the arms and coming with a few inches off the ground in every repetition. The test was modified for females who performed push-ups on their knees, whereas males performed them from the standard position with their toes making contact with the ground. Participants then entered the scores from these into the survey.

#### Work-related outcomes

The Utrecht Work Engagement Scale (UWES) was used to measure work engagement^22^. UWES-17 is made up of 17 questions scored from a 7-point Likert scale, with 0 expressing that you “never” have the feeling related to the question and 6 expressing that you “always” do. Low scores are indicative of burnout, and high scores indicate an employee’s ability to effectively deal with the demands of the job. This survey produced a total work engagement score but also has 3 sub-scales which consist of vigour, dedication and absorption. The overall score is the average of 17 questions, and the score for each sub-scale is the average of each question related to that sub-scale. A total score of ≤ 1.93 is considered “very-low”, and a total score of ≥ 5.54 is considered “very high”^23^.

The Perceived Stress Scale (PSS) was used to measure participants’ stress in the prior month^24^. This scale consists of 10 questions answered using a five-point Likert scale from 0 (never) to 4 (very often) and produces one total score. Higher scores indicate higher levels of stress; scores of 0–13 are considered low, 14–26 is considered moderate, and 27–40 is considered high stress^25^.

The Health and Work Questionnaire (HWQ) was used to measure the health and productivity of employees^26^. The HWQ consisted of 24 questions used to create an overall HWQ score and six subscales: productivity, concentration/focus, supervisor relations, impatience/irritability, work satisfaction and non-work satisfaction. Scoring was on a Likert scale from 1 (worse productivity) to 10 (best productivity), therefore, high scores relate to higher productivity. Scores for each subscale are calculated from the sum of responses to all the items comprising the subscale divided by the number of items in the subscale. No normative HWQ values have been reported.

#### Recruitment and adherence rates

Due to the broad recruitment approach, it was not possible to determine recruitment rates as the overall reach of these strategies is unknown. Adherence to the intervention was recorded via weekly check-in emails, where participants were asked to report whether they had completed all three sessions. Any adverse harms were also reported via check-in emails. 

### Statistical analysis

Survey data was exported to Excel version 15.x-16.16 (Microsoft), and all statistical analysis was performed using SPSS version 28.0 software (SPSS Inc., Chicago, IL). A sample size of 30 was determined based on guidelines for feasibility studies by Julious^27^ suggesting a minimum of 12 participants per arm, and allowing for a rate of 20% attrition. No further formal sample size calculations were undertaken due to this being a feasibility study.

Data was visualised using box plots to check for outliers and then checked for normal distribution. Any missing data was imputed using the corresponding answer given in their baseline survey answer (last-observation-carried-forward)^28^. Demographic data gathered at baseline were cleaned to condense the number of categories where necessary, so that no category had fewer than 5 people in it. Continuous variables are presented as mean ± *SD*, and categorical variables as *n* (%). A 2 × 2 mixed ANOVA was conducted to examine the group x time interaction effects on physical function tests and work-related outcomes. All analyses were intention-to-treat. Assumptions were checked for all statistical analyses, and significance was set at *p* < 0.05. Adherence was calculated as the percentage of self-reported sessions completed compared to those expected (12 expected sessions).

## Results

**Fig. 1:**
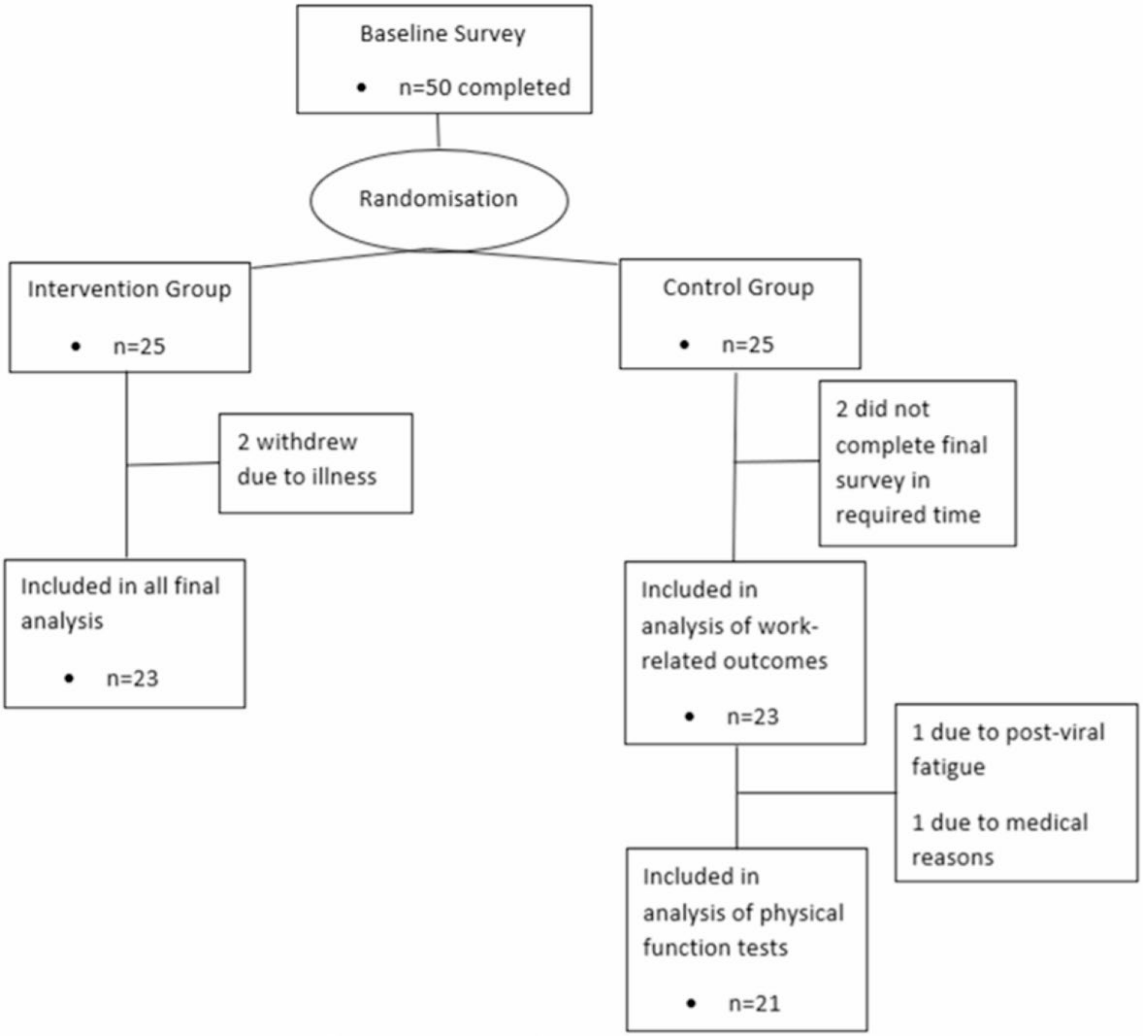
Flow chart of participants during the trial.

The CONSORT flowchart is shown in Fig. 1. Seventy-five people responded to the recruitment strategies. Whilst it was not possible to calculate true recruitment rates, the study exceeded initial recruitment targets in 1 week. Twenty-five were excluded for not meeting the initial eligibility criteria, and two were excluded after consenting based on their responses to the PAR-Q+. Fifty people completed baseline measures. Of these, 92% (*n* = 46) completed the final survey and were included in the final analysis for all work-related outcomes, with *n* = 44 completing the physical function tests.

Demographic information is described in Table 2. The sample consisted mainly of well-educated (80% university degree or higher), white (96%), females (82%) with a mean age of 46.38 ± 11.19 years (range 23–65). No harms or adverse events were reported during the trial. Adherence for the intervention group expressed as a total number of sessions completed was 89%.

**Table 2:** Participant characteristics at Baseline.

Variable	Total (*n* = 50)	Intervention Group (*n* = 25)	Control Group (*n* = 25)
Age (mean ± *SD*, years)	46.38 ± 11.19	44.48 ± 11.76	48.28 ± 10.48
Female, *n* (%)	41 (82)	20 (80)	21 (82)
Ethnicity, *n* (%)			
White	48 (96)	25 (50)	23 (46)
Other	2 (4)	0 (0)	2 (4)
Scottish Index of Multiple Deprivation, *n* (%)			
1–3	24 (48)	14 (28)	10 (20)
4–5	25 (50)	11 (22)	14 (28)
Education, *n* (%)			
University or Higher	40 (80)	19 (38)	21 (42)
No University Degree	10 (20)	6 (12)	4 (8)
Employment Status, *n* (%)			
Full-time	44 (88)	22 (44)	22 (44)
Part-time	6 (12)	3 (6)	3 (6)
Annual Income, *n* (%)			
Less Than GBP 30,000	18 (36)	8 (16)	10 (20)
GBP 30,001 to 40,000	11 (23)	6 (12)	5 (10)
Over GBP 40,001	19 (38)	10 (20)	9 (18)
Work Sector, *n* (%)			
NHS	25 (50)	11 (22)	14 (28)
Other	25 (50)	14 (28)	11 (22)
Expected work hours (mean ± *SD*)	34.41 ± 6.80	34.82 ± 6.86	34.00 ± 6.87
Hours Completed in Previous Week (mean ± *SD*)	36.40 ± 9.47	36.92 ± 9.15	35.88 ± 9.95
Perceived Health, *n* (%)			
Poor/Fair/Good	29 (58)	15 (30)	14 (28)
Very Good/Excellent	21 (42)	10 (20)	11 (22)

Changes in physical function tests and work-related outcomes are shown in Table 3. There was a significant group x time interaction effect for the 30 s sit-to-stand test scores (5.34 ± 7.88, 95% *CI* [1.94, 8.75]; *p* = 0.04) and 30 s push-up test scores (5.00 ± 3.37, 95% *CI* [3.54, 6.46]; *p* < 0.001).

For PSS, there was a significant group x time interaction effect (*p* = 0.002) with a mean reduction in stress of −5.17 ± 6.54 for the intervention group. For UWES, there was a significant group x time interaction effect for total work engagement (*p* = 0.008) and the three work engagement sub-scales of vigour (*p* = 0.001), dedication (*p* = 0.04) and absorption (*p* = 0.05). There was a significant group x time interaction effect for total HWQ score (*p* = 0.004) and the subscales of non-work and work satisfaction (*p* = 0.008 and *p* = 0.001), but not for any of the other subscales (productivity, concentration, supervisor relations and impatience).

**Table 3:** Changes in physical function tests and work-related outcomes.

Measure	Control Group, *n* = 23 (mean ± SD)			Intervention Group, *n* = 23 (mean ± SD)			*p*-value (ANOVA)
	Baseline	Post-Intervention	Mean Change (95% CI)	Baseline	Post-Intervention	Mean Change (95% CI)	
30 s Sit-to-Stand Test	15.65 ± 4.54	17.17 ± 5.67	1.52 ± 3.76 (−0.10 to 3.15)	15.65 ± 2.92	21.00 ± 6.78	5.34 ± 7.88 (1.94 to 8.75)	0.04*
30 s Push-up Test	11.52 ± 4.24	12.60 ± 4.80	1.09 ± 2.02 (0.21 to 1.96)	12.00 ± 4.81	17.00 ± 4.87	5.00 ± 3.37 (3.54 to 6.46)	< 0.001*
PSS	14.70 ± 6.06	15.04 ± 6.52	+ 0.35 ± 4.90 (−1.77 to 2.47)	16.35 ± 5.92	11.17 ± 4.90	−5.17 ± 6.54 (−8.00 to −2.35)	0.002*
HWQ Total	7.07 ± 1.51	7.14 ± 1.50	0.07 ± 0.96 (−0.35 to 0.48)	7.14 ± 0.96	8.02 ± 0.95	0.88 ± 0.87 (0.50 to 1.25)	0.004*
Productivity	7.00 ± 1.60	6.89 ± 1.42	−0.11 ± 1.11 (−0.60 to 0.38)	7.04 ± 1.23	7.57 ± 0.93	0.53 ± 1.06 (0.07 to 1.00)	0.06
Concentration	6.30 ± 2.41	7.10 ± 2.24	0.80 ± 1.80 (0.02 to 1.58)	6.40 ± 1.95	7.91 ± 1.59	1.51 ± 1.42 (0.90 to 2.13)	0.14
Supervisor Relations	6.54 ± 2.68	6.98 ± 2.47	0.43 ± 1.80 (−0.34 to 1.21)	7.24 ± 1.76	8.33 ± 1.50	1.09 ± 1.94 (0.25 to 1.93)	0.24
Non-Work Satisfaction	8.13 ± 1.34	7.74 ± 1.51	−0.39 ± 1.41 (−1.00 to 0.22)	8.19 ± 1.34	8.86 ± 0.89	0.67 ± 1.67 (0.16 to 1.17)	0.008*
Work Satisfaction	6.96 ± 1.86	6.96 ± 1.93	0.00 ± 1.29 (−0.56 to 056)	6.91 ± 1.30	8.21 ± 1.07	1.30 ± 1.30 (0.74 to 1.86)	0.001*
Impatience	7.84 ± 1.69	8.00 ± 1.66	0.19 ± 1.42 (−0.42 to 0.80)	8.30 ± 1.53	8.49 ± 1.90	0.16 ± 1.82 (−0.63 to 0.95)	0.95
UWES Total	3.79 ± 1.20	3.74 ± 0.95	−0.05 ± 0.57 (−0.30 to 0.19)	3.82 ± 0.86	4.29 ± 0.86	0.47 ± 0.70 (0.17 to 0.78)	0.008*
UWES Vigor	3.60 ± 1.12	3.60 ± 0.96	0.00 ± 0.55 (−0.24 to 0.24)	3.62 ± 0.83	4.28 ± 0.77	0.67 ± 0.77 (0.34 to 1.00)	0.001*
UWES Dedication	4.06 ± 1.37	3.96 ± 1.13	−0.10 ± 0.66 (−0.39 to 0.18)	4.10 ± 1.07	4.44 ± 1.21	0.35 ± 0.76 (0.01 to 0.68)	0.04*
UWES Absorption	3.76 ± 1.28	3.70 ± 0.88	−0.07 ± 0.75 (−0.39 to 0.26)	3.78 ± 0.90	4.17 ± 0.86	0.38 ± 0.79 (0.04 to 0.72)	0.05*

*SD* = Standard Deviation, *CI* = Confidence Interval.

*p*-value for mixed ANOVA group x time interaction.

*Denotes statistical significance, *p* < 0.05.

## Discussion

The results of this study suggest that a 4-week at-home resistance band training intervention increased lower and upper body physical function. The current data also showed that the intervention resulted in significant improvements in secondary outcomes of perceived stress, work engagement and productivity. To our knowledge, this is the first trial to show improvements in both physical function and work-related outcomes from resistance band training in hybrid-working employees.

With regard to physical function tests, the results from the current study align with previous research on resistance bands and physical function. Orange et al^29^., reported a 4-week trial (total sample *n* = 36) comparing supervised to unsupervised home-based resistance band training in healthy older adults, and found an increase in lower body function measured by the 30 s sit-to-stand score in both groups. Their participants were a similar age to the current study (46.4 ± 11.2 years in the current study and 53.6 ± 3.6 years in the previous study) and reported comparable change scores (5.34 sit-to-stand in the current study, + 3.4 sit-to-stand supervised group and + 3.1 sit-to-stand unsupervised group in the previous study). Colado and Triplett^30^ conducted a 10-week randomised controlled trial of sedentary middle-aged women, with participants randomised to resistance band training (*n* = 21), weight training (*n* = 14) or control group (*n* = 10). The current study found a similar % increase compared to their resistance band group in both sit-to-stand scores (34% compared to 27%) and push-up score (42% compared to Coladom and Triplett 31% increase)^30^. Combined, these data suggest promising findings for the effects of even short periods (4 to 10 weeks) of resistance band training on improving both lower and upper body function.

For work-related outcomes, positive effects were reported for stress, work engagement and productivity. Previous research has shown that exercise interventions in general are effective for reducing perceived stress in employees. A 4-week randomised controlled trial by Bretland and Thorsteinsson^31^ (*n* = 49 total sample) found reductions in stress, as measured by the Perceived Stress Scale, in both resistance training and aerobic training groups compared to a control group. Their resistance training group had a 34% reduction in stress, which is comparable to the present trial with a reduction of 32%. The trial required > 30 min 3x per week of more intense resistance training than the bands used in the current study, suggesting a that even lower doses of resistance training can have a positive effect on stress.

The positive change in work engagement reported in the current study is supported by previous observational studies. Cross-sectional evidence with a study of 1321 employees reported that high levels of workplace exercise were associated with increased vigour and work engagement, measured by UWES^17^. However, previous interventional research using the same measure has shown that physical activity/exercise has a limited effect on work engagement in employees. For example, a 6-month randomised controlled trial with a mixed exercise intervention (resistance, aerobic exercise and yoga) in 367 employees found no effect on UWES scores^32^. The authors concluded that high compliance was an important factor for work engagement and vitality-related outcomes, recommending further exploration of work engagement and compliance/adherence. In the current study, adherence was high, with 89% completing all weekly sessions, which could explain the positive findings for this outcome.

For productivity, whilst there were significant changes in the total score for the HWQ, this was driven by the subscales related to work and non-work satisfaction which showed significant changes for the intervention group over time. Explanations for this could relate to employees feeling a better work-life balance from managing to fit in exercise into their daily routine, which before had to be fitted in outside of work hours. Whilst the specific productivity subscale didn’t increase significantly, it was positive that even with time taken out of work (~ 45 min per week), productivity did not decrease. Comparing these results with other literature is difficult, as limited studies recording productivity and physical activity using the HWQ are available. One study using the HWQ explored a pre-post 4-week workplace step-count challenge with 246 employees^33^. They reported a 5% increase in total HWQ score, whereas the present study saw a larger improvement in this score of 12%. The reasons for these differences are largely unknown but could relate to the mode and dose of activity delivered. Resistance band exercises can be adapted to increase higher intensity whilst maintaining time efficiency, whereas a step count challenge requires a significant time commitment to accrue more steps as the targets increase. For productivity, there may be a balance between taking time out of work to be active (unproductive worktime) and the benefit of increased productive worktime.

The adherence in this study was 89%, similar to the trial by Orange et al.^29^ which reported 99% adherence in the unsupervised home-based training group during a four weeks of resistance training (23 min, 3x per week). Overall, this shows that home-based resistance band training over the short term is associated with good adherence and likely related to the simplicity of the intervention and low time commitment. The long-term adherence to such home-based resistance band activities is not yet known. However, a six-month randomised controlled trial of high intensity, low volume resistance training in young overweight males with a similar time commitment (15 min, 3x per week) also showed a high adherence rate of 96% as well as significant improvements in both upper and lower body strength^34^. Whilst this intervention was of a higher intensity than resistance bands, it suggests that the time efficiency of resistance training interventions is important to adherence. Future qualitative work is needed to confirm this and explore what factors are important in sustaining engagement with muscle strengthening exercise over a longer period of time.

The strengths of this study include the randomised controlled trial design and the design of the resistance band programme itself, which was simple, time-efficient and adhered to well. The study also recruited more participants than expected and mainly females, who are a key demographic with regard to increasing strength. The study also included measures that interest workplaces (stress, work engagement and productivity), which was a strength as this provides evidence to support the benefits of such interventions at a workplace level. However, limitations included a highly educated sample, and more work is needed to explore similar interventions in more diverse groups. Other limitations of the study include a lack of blinding. Whilst blinding is not possible for participants in an exercise trial, blinding of the research team for statistical analysis and measurement should be considered. In addition, whilst video explanations and clear instructions were provided on the physical function outcome measures, they were unsupervised, therefore, correct technique and scoring of these tests were not guaranteed.

Future research should look to replicate this study on a larger scale incorporating the recommendations made in the current study, such as monitoring adherence over a longer period, improving the study design with blinding, supervising baseline physical function tests and recruiting a more diverse sample. Qualitative work should also investigate what aspects of the intervention were most appealing and potentially leading to high adherence, and what adaptations would increase its acceptability to other hybrid working populations, including men and those from lower socioeconomic status areas.

## Conclusion

Overall, our findings show that 4 weeks of home-based resistance band training improves both lower and upper body physical function, perceived stress, work engagement and productivity in hybrid-working employees. The simplicity and low time commitment may be important factors for adherence to the intervention and muscle strengthening exercises. Future research should focus on increasing the successful implementation of home-based resistance band training for a wider range of hybrid workers.

## Supplementary Information

Below is the link to the electronic supplementary material.

Supplementary Material 1

Supplementary Material 2

Supplementary Material 3

## Data Availability

Data will be made available upon reasonable request from the corresponding author.

## Abbreviations

PAR-Q+: Physical Activity Readiness Questionnaire for Everyone
UWES: Utrecht Work Engagement Scale
PSS: Perceived Stress Scale
HWQ: Health and Work Questionnaire
