# Supplementary material for: Strength training intervention for hybrid workers: a randomised pilot feasibility trial
Source: Sci Rep. 2025 Nov 25;15:41745. doi: 10.1038/s41598-025-27567-9 (PMC12647125; doi:10.1038/s41598-025-27567-9)
Supplement: Supplementary file 3 — Supplementary Material 3 [file 41598_2025_27567_MOESM3_ESM.docx]

**Research Protocol for Strength Training Intervention for Hybrid Workers: A Randomised Pilot Feasibility Trial**

**Project Summary**

Since the COVID-19 pandemic, working practices for office-based employees have changed significantly, where working from home having increased. This change in working practices has, however, affected employees’ lifestyle behaviours and health and wellbeing. There is a major productivity and economic loss due to poor mental and physical health of the workforce. Home-based muscle strengthening interventions designed specifically for the hybrid working employee population are needed, as they can improve these factors. Therefore, this study aimed to investigate the effect of a time-efficient, resistance band training intervention for hybrid working employees on physical function and work-related outcomes.

This project is a pilot randomised controlled trial, involving untrained hybrid-working employees who will be randomised to a four-week resistance band training intervention (15 mins 3x per week) or control group. Written and video instructions on the exercises were provided. The primary outcomes were physical function; 30s sit-to-stand and 30s push-up test, with secondary outcomes of perceived stress, work engagement and productivity. Outcome data was collected via an online survey. All of the physical function tests and online surveys will be conducted remotely from the participant’s home.

This study is expected to generate evidence on the effectiveness of a low-cost, home-based resistance training intervention for improving workplace outcomes and strength. Insights may inform workplace wellness policies and health system strategies by promoting scalable, preventative approaches to physical function and well-being. The results from this study will be presented at scientific meetings, published in scientific journals and used in the research project reports of a master’s student undertaking this research. The results will be disseminated through practical resources for employers and health professionals, supporting real-world application.

**General Information**

| **Title** | Strength Training Intervention for Hybrid Workers: A Randomised Pilot Feasibility Trial |
| --- | --- |
| **Date** | 6 April 2022 |
| **Sponsor/Funding** | Internal funding for University of Glasgow MSc Sports, Exercise and Sports Medicine Projects |
| **Investigators** | Christopher Connelly, MSc, School of Sport, Exercise and Health Sciences, Loughborough University  Gemma C Ryde, PhD, School of Cardiovascular and Metabolic Health, University of Glasgow  Stuart Gray, PhD, School of Cardiovascular and Metabolic Health, University of Glasgow  Nur Dania Rosaini, BSc, School of Cardiovascular and Metabolic Health, University of Glasgow |
| **Location and/or Institution Involved** | Participants will perform the exercise intervention from home and also perform functional tests (which an outcomes) from home. University of Glasgow is the institution involved. |

**Rationale and Background Information**

The role of strengthening activities in improving and maintaining health are well known and include reducing muscle loss (both quality and quantity), falls prevention and bone strengthening (Warburton et al. 2017). However, only 31% of men and 24% of women in the UK meet both the aerobic and strength/resistance guidelines (Scottish Health Survey 2017). Many barriers to resistance training have been reported, including time constraints and access to a gym. With the shift to home/hybrid working, home-based resistance training programs using resistance bands, which can be purchased at a low cost, and body weight exercises may be an effective strategy to help increase activity levels.

To date, resistance band programs in home-working adults is yet to be explored. Positive effects of similar programs have been reported in different populations, such as older adults (Orange et al. 2019) and can produce similar benefits to weight training (Lopes et al. 2019). Outcomes of interest to workplaces, such as burnout and stress have can also be positively influenced, but this has not yet been tested in home-based employees. Therefore, a low-volume, home-based resistance training intervention will be developed and investigated to see if this can have positive benefits to work-related outcomes and strength.

Reference:

- Warburton DER, Bredin SSD. Health benefits of physical activity: a systematic review of current systematic reviews. Curr Opin Cardiol. 2017 Sep;32(5):541-556. doi: 10.1097/HCO.0000000000000437. PMID: 28708630.
- The Scottish Government. Scottish Health Survey 2018: main report - revised 2020 2020 [Available from: <https://www.gov.scot/publications/scottish-health-survey-2018-volume-1-main-report/>
- Orange ST, Marshall P, Madden LA, Vince RV. Short-Term Training and Detraining Effects of Supervised vs. Unsupervised Resistance Exercise in Aging Adults. J Strength Cond Res. 2019;33(10):2733-42.
- Lopes JSS, Machado AF, Micheletti JK, de Almeida AC, Cavina AP, Pastre CM. Effects of training with elastic resistance versus conventional resistance on muscular strength: A systematic review and meta-analysis. SAGE Open Med. 2019;7:2050312119831116.

**Study Goals and Objective**

The aims of this study are to:

- Develop a low-volume, low-cost home-based resistance training intervention
- Compare the effects of the intervention to a control on workplace outcomes of stress, productivity, work performance and work engagement
- Compare the effects of the intervention to a control on musculoskeletal health
- Compare the effects of the intervention to a control on physical function/strength

**Study Design**

| **Study Design** | This study will utilise Randomised Control Trail (RCT) design. Following completion of the baseline measurements participants will be randomised (stratified by sex) to either a waiting list control or exercise intervention group. The control group will be asked to maintain their habitual activity and will be offered the intervention at the end of the study. Those in the exercise intervention group will all take part in a home-based low volume resistance exercise intervention. All participants will be asked to maintain their normal dietary habits throughout the study. |
| --- | --- |
| **Participants and Recruitment** | Participants will be adults (16 year and older) working from home at least 3 days per week and not currently achieving government strength guidelines of 2 days per week. Participants will not be eligible for this study if they are unable to exercise and will be excluded if they have had prior history of heart, lung, cancer, kidney, endocrine, or liver disease. Participants must pass the physical activity readiness questionnaire (PAR-Q) to be eligible to join the study. Invites to participate in the study will be sent out by the research team through their existing social media channels (Twitter, Facebook) and existing workplace contacts. |
| **Sample Size** | The current study is a pilot RCT study and no formal sample size calculation has been carried out. The main component of the intended work is to collect preliminary data to inform the sample size calculations for a subsequent definitive trial. Sample size is therefore based on guidelines from Julious (2005) suggesting a minimum of 12 participants per group, with allowance for a 50% drop out per group, meaning 50 employees will be recruited (25 people per group). In order to get a gender balance, males’ participants will be capped at 25 and the recruitment strategy adapted to ask for women only.  Reference: Julious SA. Sample size of 12 per group rule of thumb for a pilot study. Pharm Stat. 2005;4(4):287-91. |
| **Intervention and Development** | The intervention will take place over 4 weeks. The employees will be provided with a set of resistance bands free of charge (~£15). Each person will start with the same bands for the prescribed exercises in week 1. They will be given a range of reps to achieve for each session (8-12). The prescribed band for the 2nd week will be based on the number of reps achieved in the previous week and perceived effort for each exercise. The exercises will be performed once per day 3x/week on days chosen by the participants, with 2 sets in weeks 1 and 2 and 3 sets in weeks 3 and 4. A similar protocol was shown to have positive effects on adults aged 50+ (Orange et al. 2019). The following exercise will be performed: lateral walk, squat, glute raise, deadlift, modified press-up, standing scapula retraction, seated row, core rotation. Exercises will be paired up by muscle group so that participants can complete another exercise whilst another muscles group is resting (e.g. squats and modified press-up). Exercises will be recorded in a short video introduction that participants can watch and rewatch to ensure they are doing them correctly. A project steering group will be created consisting of colleagues and existing contacts of the research team who will refine the final intervention to be delivered. This is largely in relation to the type of activities to be included to ensure they are suitable for performing in work clothes and without needing to shower. Adjustments are likely to include switching of exercises or performing fewer reps and sets – and not increasing the activity detailed above.  Reference: Orange ST, Marshall P, Madden LA, Vince RV. Short-Term Training and Detraining Effects of Supervised vs. Unsupervised Resistance Exercise in Aging Adults. J Strength Cond Res. 2019;33(10):2733-42. |

**Methodology**


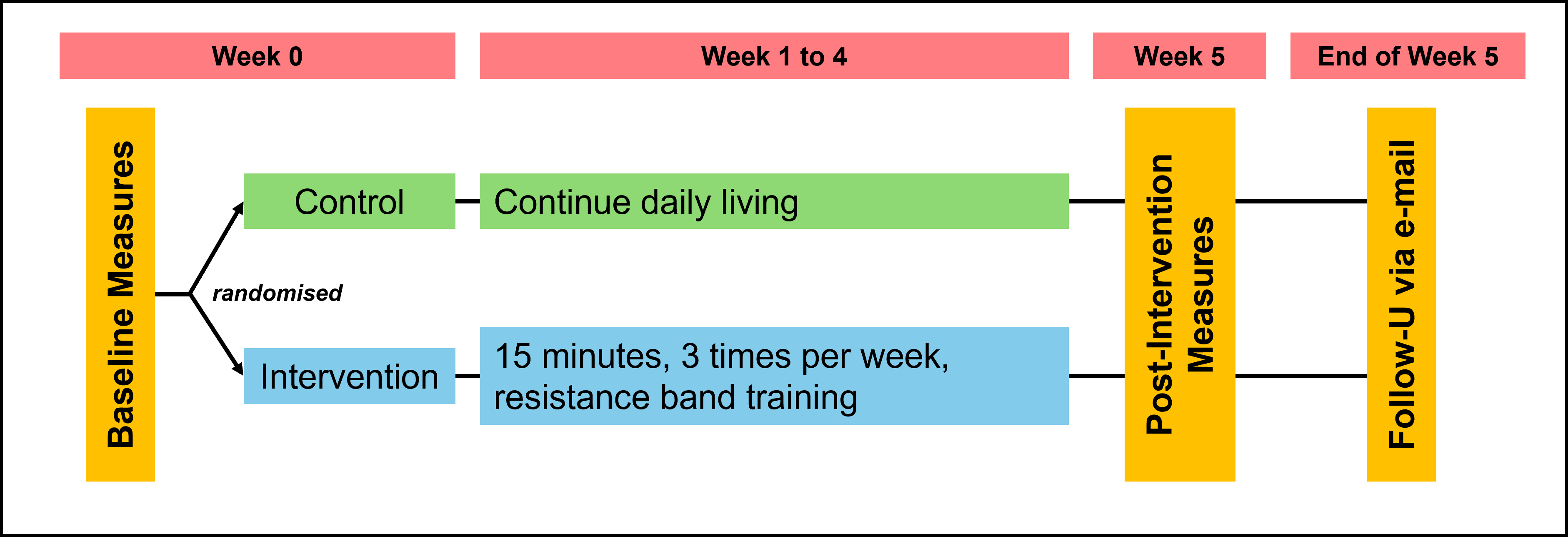


| **Overview** | Data will be collected through an online survey and physical function tests. For the function tests, participants will either be provided with a video instruction or attend a live, online session with a researcher to see how to preform and score the tests. They will be asked to score their own tests and either email these to the researchers or enter the details into the online survey. All measures will be collected at baseline and after the 4 weeks. Each test/measure are detailed further below. |
| --- | --- |
| **Online Survey** | The survey will include questions on demographics (age, gender, ethnics background, postcode, qualification, general health), Job (employment status, job title, annual income, working from home status, hours worked, physical activity at work), work related outcomes (health related quality of life - HRQL, stress - Perceived Stress Scale, productivity - HWQ, work performance - HPQ short, work engagement - UWES), physical activity (PA levels -GPAQ, days of strength training) and musculoskeletal conditions (Roland-Morris Disability Questionnaire, the Keele STarT Back Screening Tool). In the follow up survey, no demographic data will be collected and instead, open ended questions will be provided for employees to feedback on any improvements or changes to the intervention and protocol. |
| **Push-Up Test** | The test requires participants to complete the maximal amount of push-ups consecutively without rest. Participants will either start on their knees if female or in the standard down position for males. The test will stop when the participant fails to reach the appropriate technique on two consecutive attempts. |
| **30-Second Sit to Stand Test** | The participant is encouraged to complete as many full stands as possible within 30 seconds. As participants will be at home, a stable dining chair will be recommended for the test. The number of stands a person can complete in 30 seconds is counted as the score. |
| **Participation** | Exercise adherence will be measured through weekly check-ins by email asking the participants to report the actual dose achieved compared to the dose prescribed. |

**Safety Consideration**

Exercise has a negligible risk in healthy adults, although maximal exercise does carry an extremely small risk of inducing myocardial ischaemia (“heart attack”). This program is low-volume and submaximal. Exercise will be at a submaximal level at most points in the study, but there is the possibility that the subject group will suffer from acute muscle soreness. Participants will not be eligible for this study if they are unable to exercise and will be excluded if they (i) have undergone surgery for weight loss; (ii) have had a prior history of heart, lung, cancer, kidney, endocrine, or liver disease. The risks associated with participating in this study are very small. It is more likely that the participants will gain benefit from the study than not. It will also provide evidence of the benefits of this sort of program for home workers which is essential given the low numbers of people adhering to the strength exercise guidelines

**Follow-Up**

Follow-up (via email check-in) for adverse events will be conducted post-intervention with data collection at the end of week 5. Participants will be allowed to keep the bands if they complete the follow-up data collection and respond to weekly adherence emails.

**Data Management and Statistical Analysis**

| **Data Management** | Data will be collected from of an online survey and functional fitness tests. Both qualitative and quantitative data will be produced from the online survey which will be exported in the form of an Excel spreadsheet. Quantitative data will be transferred from the Excel spreadsheet and analysed in the data analysis package SPSS. Qualitative data from the survey will be exported into NVivo. Fitness test data produces a score which will either be recorded by the researcher for live, online fitness test, or entered into the survey for those completing the tests in their own time.  Only the named investigators will have full access to the data obtained. The information obtained will be anonymised and identifiable data will not be passed on to anyone outside the named investigators. Data will be stored on personal or University of Glasgow computers of the named investigators on the University server. Access will be restricted by password-protection and data will be stored for 10 years. Data will be backed up through the University of Glasgow OneDrive cloud. |
| --- | --- |
| **Statistical Analysis** | Survey data will be exported to Excel (2016), and all statistical analysis will be performed using SPSS version 28.0 software (SPSS Inc., Chicago, IL). A sample size of 30 was determined based on guidelines for feasibility studies by Julious (2005) suggesting a minimum of 12 participants per arm, and allowing for a rate of 20% attrition. No further formal sample size calculations were undertaken due to this being a feasibility study.  Data will be visualised using box plots to check for outliers and then checked for normal distribution. Any missing data will be imputed using the corresponding answer given in their baseline survey answer (last-observation-carried-forward). Demographic data that will be gathered at baseline will be cleaned to condense the number of categories where necessary, so that no category had fewer than 5 people in it. Continuous variables will be presented as mean ± SD, and categorical variables as n (%). A 2x2 mixed ANOVA will be conducted to examine the group x time interaction effects on physical function tests and work-related outcomes. All analyses will be treated as intention-to-treat. Assumptions will be checked for all statistical analyses, and significance will be set at p<0.05. Adherence will be calculated as the percentage of self-reported sessions completed compared to those expected (12 expected sessions). |

**Quality Assurance**

The lawful basis for processing, as outlined in the privacy notice (pages 12-13), is based on consent of the participant. They are consenting to inclusion of their health data. The processing of the data will ensure an analysis is performed and ultimately help to create an exercise program that can be promoted to others. This outcome cannot be achieved without conducting this research. Function creep will be prevented by only asking essential questions in the survey and restricting the functional fitness tests. Individuals will be given an information sheet detailing the study and can request a copy of their data at any time and withdraw from the study. A report of the study can be provided upon request.

**Expected Outcomes of The Study**

This study will generate evidence on the effectiveness of a low-cost, home-based resistance training intervention for improving workplace outcomes and strength. Insights may inform workplace wellness policies and health system strategies by promoting scalable, preventative approaches to physical function and well-being.

**Dissemination of Results and Publication Policy**

The results from this study will be presented at scientific meetings and published in scientific journals. The results will also be used in the research project reports of a master’s student undertaking this research. The results will be disseminated through practical resources for employers and health professionals, supporting real-world application.

**Duration of the Project**

| **Month** | **Task** |
| --- | --- |
| September 2021 | Project Planning |
| April 2022 | Ethic Application |
| May 2022 | Recruitment |
| May-July 2022 | Data Collection |
| July 2022 | Data Analysis |
| July-August 2022 | Write-Up |

**Problems Anticipated**

Here are the problems anticipated, and the solution planned.

1. Recruitment and potential dropout – Longer recruitment period and potentially paid advertising on social media
2. More males than females – stop recruitment when half sample is males.
3. Potential Covid influence – delivery data collection remotely as planned

**Project Management**

| **Gemma Ryde** | Conceptualisation, Methodology, Formal Analysis, Writing - Original Draft, Writing - Review & Editing, Visualisation, Supervision, Project Administration. |
| --- | --- |
| **Christopher Connelly** | Investigation, Data Curation, Writing - Original Draft, Writing - Review & Editing, Visualisation, Project Administration. |
| **Stuart Gray** | Writing - Review & Editing, Visualisation. |
| **Nur Dania Rosaini** | Writing - Review & Editing, Visualisation. |

**Ethics**

| **Ethical Consideration** | An ethical consideration is that the resistance training intervention will not be offered to the control group initially; however, after the trial period of 4 weeks, they will be offered the materials to complete the intervention. |
| --- | --- |
| **Consent** | All subjects will be emailed the information sheet and a consent form outlining the testing procedures, which asks them for their written consent to participate in the project with the option to withdraw at any time (see enclosed copy). Consent forms will be sent back by emails. A verbal explanation will also be given and any queries answered on request. A health questionnaire will also be given (PARQ). If there is any doubt of a subject’s eligibility for the study, that subject will be excluded. |

**Informed Consent Forms**

Informed consent form is attached on pages 10-11.

**Budget**

| **Item** | **Purpose** | **Price Per Unit** | **Unit Needed** | **Total Cost** |
| --- | --- | --- | --- | --- |
| Bands | For intervention delivery | £15.00 | 50 | £750.00 |
| Postage | To send out bands | £40.00 | - | £40.00 |
| **Total Cost** | | | | £790.00 |

**Other Support for The Project**

No funding was received, and the study was funded internally.

**Curriculum Vitae of Investigators**

The curriculum vitae of investigators is attached on pages 14-18.

***
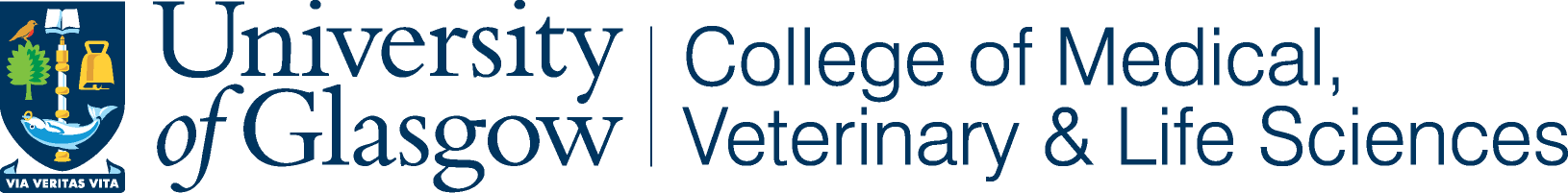
***

|  |  | | | | |
| --- | --- | --- | --- | --- | --- |
| Project Number: |  | | | | |
| Participant Identification Number: | | | |  | |
| **Title of Project:** | | Resistance training in home working employees | | | |
| **Name of Researcher(s):** | | | Dr Gemma Ryde, Mr Christopher Connelly | | |
|  | | | | | |
| **CONSENT FORM** | | | | | Please initial box |
| **I confirm that I have read and understood the Participant Information Sheet version 1 dated 06/04/2022.** | | | | |  |
| **I confirm that I have read and understood the Privacy Notice version 1 dated 06/04/2022.**  **I have had the opportunity to think about the information and ask questions and understand the answers I have been given.** | | | | |  |
| **I understand that my participation is voluntary and that I am free to withdraw at any time, without giving any reason, without my legal rights being affected.** | | | | |  |
| **I confirm that I agree to the way my data will be collected and processed and that data will be stored for up to 10 years in University archiving facilities in accordance with relevant Data Protection policies and regulations.** | | | | |  |
| **I understand that all data and information I provide will be kept confidential and will be seen only by study researchers and regulators whose job it is to check the work of researchers.** | | | | |  |
| **I agree that my data described in the information sheet will be kept for the purposes of this research project.** | | | | |  |
| **I understand that if I withdraw from the study, my data collected up to that point will be retained and used for the remainder of the study.** | | | | |  |
| **I agree to take part in the study.** | | | | |  |

#### Name of participant Date Signature

#### Name of Person taking consent Date Signature

(if different from researcher)

#### Researcher Date Signature

(1 copy for participant; 1 copy for researcher)

**Privacy Notice: Resistance training in home working employees**

**Your Personal Data**

***The University of Glasgow*** *will be what’s known as the ‘Data Controller’ of your personal data processed in relation to this study. This privacy notice will explain how The University of Glasgow will process your personal data.*

**Why we need it**

*We are collecting your basic personal data such as name, contact details and health data in order to develop and evaluate a home-based resistance training program. Your name and contact details will only be used when collecting the data and will be destroyed once all data is collected.*

**Legal basis for processing your data**

*We must have a legal basis for processing all personal data. In this instance, the legal basis is consent. We must have consent to use your health and fitness data as part of this research. As your health data is sensitive personal information, we are unable to use this information without prior consent from you.*

**What we do with it and who we share it with**

*All the personal data you submit is processed by researchers at the University of Glasgow in the United Kingdom. This data will be stored securely on the University server. As stated once all data is collected your contact details will be deleted at this point. Any data collected will be given a unique ID number which belongs to you but will not be linked to your name. Therefore, your personal data will be anonymous to all other researchers.*

**How long do we keep it for**

*Your contact details will be kept for a month until we have recruited people from the survey to the interview at which point they will be deleted. All other anonymous data will be kept for 10 years.*

**What are your rights?***

*You can request access to the information we process about you at any time. If at any point you believe that the information we process relating to you is incorrect, you can request to see this information and may in some instances request to have it restricted, corrected or, erased. You may also have the right to object to the processing of data and the right to data portability.*

*If you wish to exercise any of these rights, please contact* [*dp@gla.ac.uk*](mailto:dp@gla.ac.uk)*.*

*Please note that the ability to exercise these rights will vary and depend on the legal basis on which the processing is being carried out.

**Complaints**

If you wish to raise a complaint on how we have handled your personal data, you can contact the University Data Protection Officer who will investigate the matter.

Our Data Protection Officer can be contacted at [dataprotectionofficer@glasgow.ac.uk](mailto:dataprotectionofficer@glasgow.ac.uk)

If you are not satisfied with our response or believe we are not processing your personal data in accordance with the law, you can complain to the Information Commissioner’s Office (ICO) <https://ico.org.uk/>

􀕿 I consent to the University processing my personal data for the purposes detailed above.

􀕿 I have read and understand how my personal data will be used.

Signed: …………………………………………………………………………………………………………………

Date: ……………………………………………………………………………………………………………………

**Dr Gemma Cathrine Ryde CV**

**Academic and Professional Qualifications**

2013 **The University of Queensland** – PhD in Physical Activity and Health

Thesis: *Describing and influencing occupational physical activity and sedentary behaviour*

(Supervisors - Prof Wendy Brown and Dr Nicholas Gilson)

2005 **Heriot Watt University –** BSc Hons 1^st^ Biology with Sport and Exercise Science

**Recent Career History**

2021-current **Lecturer in Physical Activity and Health,** University of Glasgow, Scotland

2018 – 2021 **Lecturer in Physical Activity and Health,** University of Stirling, Scotland

2016-2018 **Post-Doctoral Impact Fellowship**, University of Stirling, Scotland

*Project: Describing and influencing occupational physical activity and sedentary behaviour*

**Recent Funded Research Grants**

| Hennessy C, Dawson A, Whittaker A, **Ryde GC,** Coffee P, Haynes R, Watchman K, Mannion G. Connectivity and Digital Design for Health and Well-being Across Generations, Places and Spaces. *ESRC.* 2021-2024: **£1,618,597**  Whittaker A, **Ryde GC,** Connelly J, Tomaz S, Coffee P. COVID 19 social distancing effects on social engagement, loneliness, wellbeing and behaviour. *Chief Scientist Office, Scottish Government.* 2020: **£52,289.** |
| --- |

**Recent Peer Reviewed Articles**

Swales B, **Ryde GC**, Whitaker A. A Randomised Controlled Feasibility Trial Evaluating a Resistance Training Intervention with Frail Older Adults in Residential Care: The Keeping Active in Residential Elderly (KARE) Trial JAPA.202. *Journal of Aging and Physical Activity*. 2021; 11:1-25.

Tomaz S, Coffee P, **Ryde GC**, Swales B, Neely K, Connelly J, Kirkland A, McCabe L, Watchman K, Andreis F, Martin J, Pina I, Whittaker A. Loneliness, wellbeing, and social activity in Scottish older adults resulting from social distancing during the Covid19 pandemic.  *International Journal of Environment Research and Public Health*. 2021; 18 (9), Art. No.: 4517

Niven A, **Ryde GC**, Wilkinson G, Greenwood C, Gorely T. The effectiveness of an annual nationally delivered Workplace Step Count Challenge on changing step counts across four years of delivery.  *International Journal of Environment Research and Public Health*. 2021; 18(10):5140.

Metcalfe RS, Atef H, Mackintosh K, McNarry M, **Ryde GC**, Hill DM, Vollaard NBJ. Time-efficient and computer-guided sprint interval exercise training for improving health in the workplace: A randomised mixed-methods feasibility study in office based employees. *BMC Public Health*. 2020. 20, Art. No.: 313.

**Ryde GC,** Atkinson P, Stead M, Gorely T, Evans J. Physical activity in paid work time for desk-based employees: A qualitative study of employers’ and employees’ perspectives. *BMC Public Health*. 2020; 20 (1), Art. No.:460

**Ryde GC,** Dreczkowski G, Gallagher I, Chesham RA, Gorely T. Device-measured desk-based occupational sitting patterns and stress (hair cortisol and perceived stress). *International Journal of Environment Research and Public Health*. 2019; 16(11):1906.

**Stuart Robert Gray**

**Present Appointment**

August 2022 – Present Professor of Muscle and Metabolic Health, School of Cardiovascular and Metabolic Health,

**Fellowships**

Fellow of the Higher Education Academy, awarded in 2017

Elected member of the RSE Young Academy of Scotland (2014 – 2019)

**Membership of Journal Editorial Boards**

Associate editor – Journal of Sports Sciences (2016 – present)

Associate editor - Diabetes & Metabolic Syndrome: Clinical Research & Reviews (2022 – present)

**Membership of External Committees**

Elected member of the Physiological Society Finance Committee (2013 - 2017)

Elected member of the Nutrition Society Scottish Section Committee (2017 – 2021)

Elected member of BBSRC pool of experts, committee A (2016 – 2026)

Invited research assessor for the Carnegie Trust (2018 – ongoing)

Member of BBSRC ALERT Grant panel (2021 - ongoing)

Appointed to Dunhill Medical Trust Research Grants Committee (2022 – ongoing)

**Prizes and Awards**

Pfizer Prize winner for oral presentation at The Physiological Society Main Meeting (Bristol) 2005.

Teaching Excellence Award 2016

Nominated for Best College Teacher – MVLS 2022

**Visiting Professor**

Visiting Professor to the Dasman Diabetes Institute (2021 – ongoing)

**Administrative/other roles**

Appointed member of Senate (2021- )

Member of ATHENA Swan self assessment team (2019-)

**Teaching**

Deputy Director of Education (PGT) (2022 - present)

Co-ordinator of MSc course ‘Nutritional Aids for Exercise Performance’ (20 credits) online and on-campus

Co-ordinator of SESM MSc projects online (60 credits)

Contribute teaching delivery across several PG and UG courses. I also supervise 2 4th year UG project students, ~12 MSc projects students on-campus and 4 online each year (~80 hours).

**Postgraduate Research Student Supervision**

13 completed PhD and MSc (by research) students and 9 current PhD and MSc (by research) students

**Full papers published in Peer-Reviewed Journals**

Published 167 papers, cited >10000 times, H-index: 54.

https://www.gla.ac.uk/schools/cardiovascularmetabolic/staff/stuartgray/#publications

**Funding Awarded**

Total peer-reviewed grant funding: £6,403,516

Selected recent grants

BHF: Rebalancing the fat content of the heart and muscles in Type 2 Diabetes £570,103 (CoI with Dr Dana Dawson)

Aker Biomarine: Synergist effects of krill oil and krill protein on muscle mass/function and muscle protein metabolism. (2022-2026) £65,000 (PI)

Chief Scientist Office: Prevention and early treatment of COVID-19 long term effects: a randomised clinical trial of resistance exercise. (2021-2023) £286,660 (Co-I with Prof C Berry)

Kappa Bioscience: The effects of Vitamin K2 on recovery after muscle damaging exercise in young and older people. (2021-2023) £171,849 (PI)

BHF: PhysicaLExercise and Mental Wellbeing Rehabilitation for Acute StrEss-induced Takotsubo Cardiomyopathy: the PLEASE Trial (2019-2022) - £298,134 (Co-I with D Dana Dawson)

MRC: Mechanisms of insulin resistance in South Asians (MIRSA): the roles of skeletal muscle microvasculature and mitochondrial metabolism (2019-2024) - £643,290 (Co-PI with Prof J Gill)

BHF: Muscle fat compartments and turnover as a determinant of insulin sensitivity £234,109 (2016-2018) (Co-I with Dana Dawson (PI))

ARUK: LIFT: Therapies for Inflammatory Rheumatic Diseases (2016-2020) £735,536 (Co-I with Neil Basu (PI))

BBSRC (New Investigator): Overcoming the blunted response to resistance exercise with fish oil to maximise the maintenance of muscle mass in older people (2012-2015) £330,684 (PI)

**Outreach Activities**

Science communication on shows such as BBC1 “Truth about fitness”, BBC2 “RIP OFF BRITAIN”, BBC Radio 4 “You and yours”, CBBC “All over the workplace” and BBC2 “Trust me I’m a doctor” alongside regular comments and contributions in the written press. I have also given public talks at Pint of Science, CaféMED, CafeScientifique, Glasgow Science Festival, Aberdeen Science Festival and European Researchers Night. I have been a mentor for Robertson Trust Scholars Scheme, given talks in local schools (Rewrite the headlines by RSE), rganised modern pentathlon event during British Science Festival coordinating several staff members and students during this event,

Mentor for secondary school pupils during ‘how to win gold’ and ‘how your body works’ and a

Participant in ‘i’m a scientist get me out of here’.

**Symposium Organisation**

ECSS 2015 – Invited session – Skeletal muscle lipotoxicity: Should we be interested?

**Conference Organisation**

Nutrition Society Spring Meeting Conference Organiser 2018

**Membership of Learned Societies**

The Physiological Society (2005 – Present)

International Society of Exercise Immunology (2011 – Present)

European College of Sports Sciences (2013 – Present)

Nutrition Society (2013 – Present)

**Christopher D Connelly**

Current Roles:

Doctoral Researcher in Neuromuscular Physiology – Loughborough University (2022-present).

Thesis title: Sarcopenia: from motor unit remodelling to motoneuronal output and muscle function.

University Teacher (casual) in Physiology, Anatomy, Sport Science, Kinesiology, Strength & Conditioning, Sport Rehab and Statistics – Loughborough University (2022-present).

Education:

BSc Physiology (First Class) – University of Aberdeen (2017-2021).

MSc Sport and Exercise Science and Medicine (Distinction) – University of Glasgow (2021-2022)

Publication:

Škarabot, J., Thomason, H., Nazaroff, B.M., **Connelly, C.D**., Valenčič, T., Ho, M.L., Tyagi, K., Beauchamp, J.A. and Pearcey, G.E., 2025. Training-induced alterations in the modulation of human motoneuron discharge patterns with contraction force. *bioRxiv*, pp.2025-06.

Conference Presentations:

Oral Presentations:

**Connelly, C.D**, Valenčič, T., Thomason, H., Piasecki, M., Pearcey, G.E., Folland, J.P. & Škarabot, J. (2024). Age-related differences in motor unit discharge rate as a function of contraction intensity. Abstract presented at the 29th Annual Congress of the European College of Sport Science, Glasgow, UK.*

**Connelly, C.D**, Thomason, H., Valenčič, T., O’Hanlon M., Jenz S.T., Pearcey, G.E., Piasecki, J., Piasecki, M., Folland, J.P. & Škarabot, J. (2025). Motor unit behaviour during isometric contractions in master power athletes. Abstract presented at the 30th Annual Congress of the European College of Sport Science, Rimini, Italy.*

**shortlisted for young investigator award.*

Poster Presentation:

**Connelly, C.D**, Thomason, H., Valenčič, T., O’Hanlon M., Jenz S.T., Pearcey, G.E., Piasecki, J., Piasecki, M., Folland, J.P. & Škarabot, J. (2024). Motor unit behaviour during isometric contractions in master power athletes (Preliminary Data). Abstract presented at Biomedical Basis of Elite Performance Conference, Nottingham, UK.

Society Memberships:

European College of Sport Science (2024-present).

Physiological Society (2024-present).

Other:

Doctoral Innovation Consultant – Loughborough University Incubator (2024).

**NUR DANIA ROSAINI**

E-mail: [nurdania.rosaini@gmail.com](mailto:nurdania.rosaini@gmail.com) | LinkedIn: [Nur Dania Rosaini](https://www.linkedin.com/in/daniarosaini/) |
ORCID: [0009-0001-8597-4378](https://orcid.org/0009-0001-8597-4378)

| **EDUCATION** | |
| --- | --- |
| **May 2023 – ongoing** | **Doctor of Philosophy**  (Sports Science/Sports Medicine)  *University of Glasgow, Scotland.*  **Thesis*:*** *Women in Strength (WISH): A Co-Creation Approach to Improve Women’s Participation in Muscle-Strengthening Exercises.*  **Supervisor:** Professor Stuart R. Gray, Dr. Gemma C. Ryde |
| **September 2022 – March 2023** | **Taught component of the MVLS Integrated PhD**  (Sports, Exercise Science and Medicine)  *University of Glasgow, Scotland.*  **Subjects:** Evidence-Based Methods and Statistics, Physical Activity and Health: Epidemiology, Mechanisms and Intervention, Exercise in Clinical Population, Physical Activity and Health: Public Health and Behaviour Change, Sports Injuries: Prevention and Rehab, Exercise Physiology. |
| **March 2020 – March 2022** | **Bachelor of Science (1^st^ Class Honours)**  (Sports Science)  *Universiti Teknologi MARA, Malaysia.*  **Thesis:** *The Effects of Short Duration Resistance Training Voluntary to Failure on Muscle Adaptations in Overweight Men.* |
| **WORKING EXPERIENCE** | |
| **September 2023 - current** | **Tutor and Demonstrator**  *MVLS College Services, University of Glasgow, Scotland.*   - Supported the delivery and evaluation of undergraduate teaching across multiple courses within the College of Medical, Veterinary and Life Sciences, focusing on laboratory-based learning and the assessment of lab skills and written reports. - Conducted practical skill assessments for the Royal Society of Biology Skill Tracker for Level 2 Human Biological Sciences students. - Lab courses include Cardiovascular System, Skeletal Muscle and Biomechanics, Integrated Systems Physiology – Respiratory System, Exercise Metabolism, Somatosensory and Reflexes Lab. |
| **PUBLICATION** | |
| Dighriri, A., Timraz, M., **Rosaini, N. D**., Aba Alkhayl, F. F., Boyle, J. G., Logan, G., & Gray, S. R. (2024). The impact of the time of day on metabolic responses to exercise in adults: A systematic and meta-analysis review. Chronobiology International, 41(11), 1377–1388. <https://doi.org/10.1080/07420528.2024.2419867>  **Rosaini, N.D**., Shafee, S.S.A., Bakar, AH.A., Mokhtar, N., Kamaruddin, H.K., Ismail, A.D. (2023). The Effect of Short-Duration Resistance Training Voluntary to Failure on Muscle Adaptation of Men in Overweight. In: Hassan, M.H.A., et al. Proceedings of the 8th International Conference on Movement, Health and Exercise. MoHE 2022. Lecture Notes in Bioengineering. Springer, Singapore. <https://doi.org/10.1007/978-981-99-2162-1_24> | |
